# All-polyethylene tibial components in distal femur limb-salvage surgery: a finite element analysis based on promising clinical outcomes

**DOI:** 10.1186/s13018-017-0555-6

**Published:** 2017-04-04

**Authors:** Fan Tang, Yong Zhou, Wenli Zhang, Li Min, Rui Shi, Yi Luo, Hong Duan, Chongqi Tu

**Affiliations:** Department of Orthopedics, West China Hospital, Sichuan University, Guoxue Xiang #37, Chengdu, 610041 Sichuan People’s Republic of China

**Keywords:** Finite element analysis, All-polyethylene tibial implant, Distal femur, Tumor knee prosthesis, Metal-backed tibial prosthesis

## Abstract

**Background:**

Whether all-polyethylene tibial (APT) components are beneficial to patients who received distal femur limb-salvage surgery lacks high-quality clinical follow-up and mechanical evidence. This study aimed to investigate the biomechanics of the distal femur reconstructed with APT tumor knee prostheses using finite element (FE) analysis based on our previous, promising clinical outcome.

**Methods:**

Three-dimensional FE models that use APT and metal-backed tibial (MBT) prostheses to reconstruct distal femoral bone defects were developed and input into the Abaqus FEA software version 6.10.1. Mesh refinement tests and gait simulation with a single foot both in the upright and 15°-flexion positions with mechanical loading were conducted. Stress distribution analysis was compared between APT and MBT at the two static positions.

**Results:**

For both prosthesis types, the stress was concentrated on the junction of the stem and shaft, and the maximum stress in the femoral axis base was more than 100 Mpa. The stress on the tibial surface was relatively distributed, which was 1–19 MPa. The stress on the tibial bone-cement layer of the APT prosthesis was approximately 20 times higher than that on the MBT prosthesis in the same region. The stress on the proximal tibial cancellous bone and cortical bone of the APT prosthesis was 3–5 times greater than that of the MBT prosthesis, and it was more distributed.

**Conclusions:**

Although the stress of bone-cement around the APT component is relatively high, the stress was better distributed at the polyethylene-cement-bone interface in APT than in MBT prosthesis, which effectively protects the proximal tibia in distal femur tumor knee prosthesis replacement. These results should be considered when selecting the appropriate tibial component for a patient, especially under the foreseeable conditions of osteoporosis.

## Background

As one of the original tibial component designs, all-polyethylene tibial (APT) components have been shown to produce excellent outcomes with long-term survival of over 90% in total knee arthroplasty (TKA) [1, 2]. However, biomechanical studies performed in the 1980s raised questions about the implants’ durability [3, 4]. Furthermore, in 2003, Faris et al. [5] published results of anatomic graduated component-APT components and showed the disastrous results of a flat-on-flat-designed APT implant. The combination of the biomechanical and anatomic graduated component studies led surgeons to choose the metal-backed tibial (MBT) component [4, 5]. Although MBT components were more expensive, it was thought that the cost would be offset by the biomechanical and technical advantages of modularity [6, 7]. In recent years, multiple studies have suggested that APT component prostheses seem to be acceptable devices with significantly better implant survival and reduced rates of early revision, postoperative infection, fracture, and tibial-component loosening than MBT component prostheses in TKA [8, 9].

As a type of reconstruction after limb-salvage resection, endoprosthetic replacement following en-block resection is currently the first-line standard treatment for distal femoral malignant tumors [10, 11]. Because of long bone segment defects after limb-salvage resection, endoprosthetic replacement differs from conventional TKA in both the biomechanics and overall implant survival [12, 13]. However, tumor knee prostheses with APT components have been rarely reported in limb-salvage surgery. Whether APT components are beneficial for patients with distal femoral tumors lacks high-quality clinical follow-up and mechanical evidence. Based on our previous, promising clinical outcome [14], three-dimensional finite element (FE) models of the reconstruction of distal femur defects with two types of tumor knee prostheses were constructed to explore the stress differences between the MBT and APT components in distal femur limb-salvage surgery.

## Methods

**Fig. 1:**
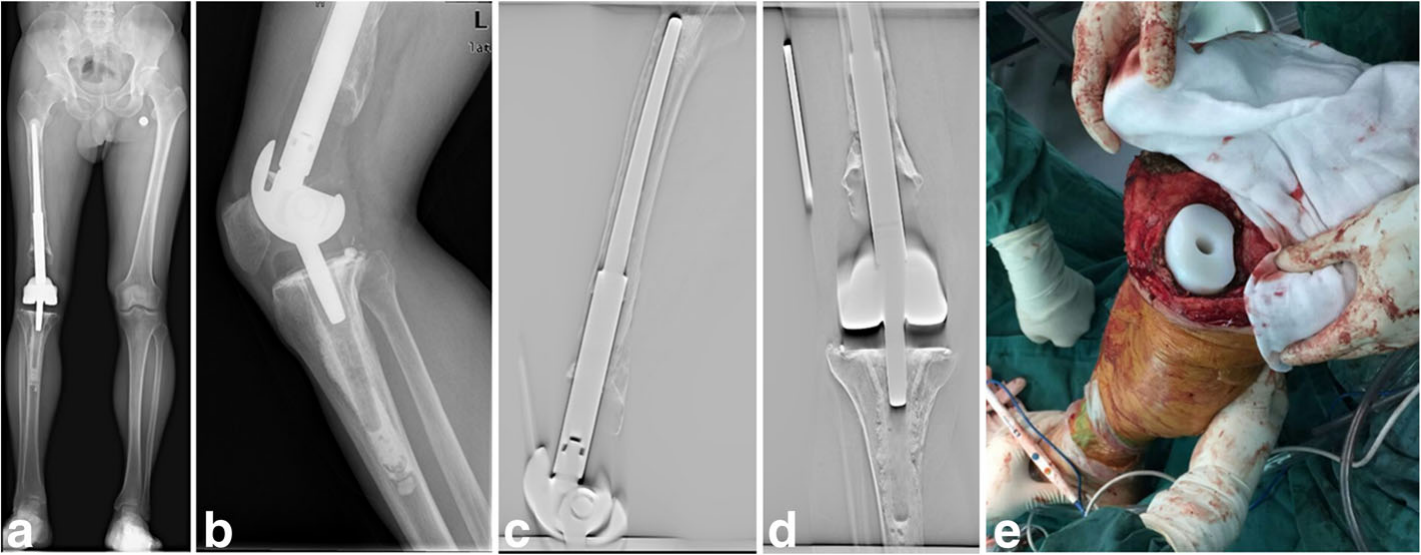
**a**, **b** X–ray of the patient who was recently underwent revision surgery, suggested aseptic loosening at the femur side 7 years after limb-salvage surgery. **c** Digital tomosynthesis (DTS) of the femur showed prosthesis stem aseptic loosening. **d** Digital tomosynthesis (DTS) of the proximal tibia showed no tibial component loosing. **e** Pictures of the APT component during revision surgery showed the APT component without obvious wear

Our department started to use APT components in tumor knee prosthesis replacement in 2006. Up to 2012, a total of 49 patients with distal femur tumors received limb-salvage surgery using APT tumor knee prosthesis replacement. Retrospectively, the mean follow-up duration of the 37 survivors was 66.3 months, and the 5-year prosthetic survival rate was 88.2%. Two prostheses were identified as aseptic loosening on the femoral side at approximately 7 years of follow-up, and one recently underwent revision surgery (Fig. 1). No stem breakage, dislocation, or peri-prosthetic fractures were observed.

### Prostheses

**Fig. 2:**
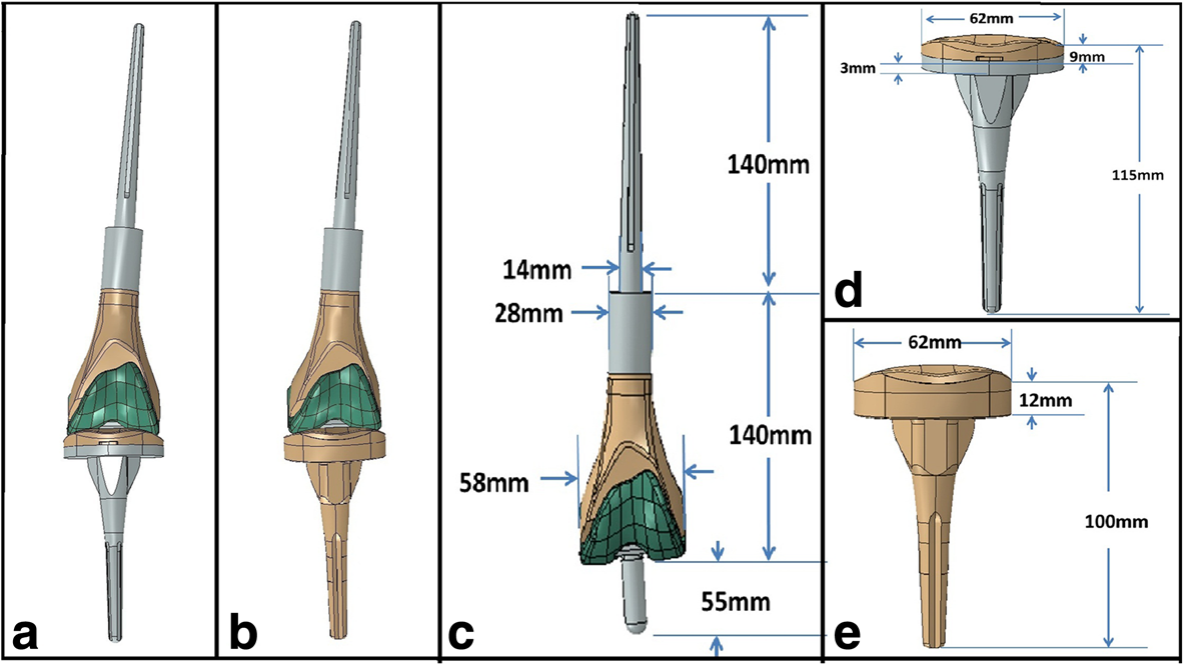
**a**–**e** Solid models of two types of prosthesis and the prosthesis parameters. **a** Solid model of meta prosthesis. **b** Solid model of polyethylene prosthesis. **c** Parameters for the femoral side. **d** Parameters for the metal-backed tibia component. **e** Parameters for the all-polyethylene tibia component

All prostheses (Chunglizhengda Medical Instruments Co., Beijing, China) were custom-made and had a rotating hinge joint. The femoral stem and tibial component were both the cement type. The prosthetic stem consisted of Ti-6Al-4V titanium alloy, the femoral condyle consisted of CO-Cr-Mo alloy, and the tibial component completely consisted of polyethylene material. The prosthesis flexion angle range was 0°–150°, and the internal or external rotation angle range was 0°–12.5° in the extension position (Fig. 2). Gentamicin bone cement (CMW®; Depuy International Ltd., Leeds, UK) was used in our study.

### Three-dimensional reconstruction models of a normal left lower limb bone and distal femoral bone defect

A healthy adult male volunteer (age, 26 years; height, 168 cm; weight, 65 kg) was selected, and his left lower limb was placed in a neutral position to undergo computerized tomography (CT) (Philips Brilliance 64CT, Philips Healthcare, The Netherlands; slice thickness = 0.7 mm with 947 slices). The scan range was from the lesser trochanter inferior margin to the middle of the tibiofibular joint. The scan parameters were as follows: 120 KV; total mAs, 1328 Ma; total dose length product (DLP), 227 mGy-cm; scanning interval, 0.06 mm; 1056 layers; length, 63.3 cm; and femoral side, 34.2 cm. The Digital Imaging and Communications in Medicine (DICOM) data of each layer were copied and recorded. Then, the DICOM data were input into Mimics 10.01 (Materialise Control Platform v10.2.1.2, Belgium); based on CT images with different gray values, the cortical bone, cancellous bone, bone marrow cavity tissue, and muscle soft tissue were defined, and the three-dimensional CT model data were obtained. The length of the resected femur ranged from 7.5 to 28 cm (mean, 14.3 cm) in our clinical follow-up patients [14]. Therefore, our study simulated a distal femoral osteotomy of 140 mm. To construct the distal femoral bone defect model, the tibia was evenly resected with a 12-mm thickness and tibiofemoral angle of 90° to the long axis of the tibia.

### Solid models of two types of prosthesis for reconstruction of the femoral distal bone defect

In the femoral distal bone defect model, the parameters of the femur stump were as follows: cortical bone thickness, 5–6 mm, and medullary cavity diameter, 16 mm. Utilizing these measurements, the prosthetic parameters of the protocol were established, as seen in Fig. 2. Using the above parameters, computer models of the two types of prostheses were generated by Chunglizhengda Medical Instruments Co. The models were placed in the “SLDASM” file format and imported into the Mimics software, which completed the three-dimensional reconstruction of the two knee prostheses. This experiment focused on the calculation of stress at the interfaces of the tumor knee prostheses so that the patellar and fibular influences were negligible. The bone defect model and knee prostheses were imported into Mimics in a three-dimensional space organization. The thickness of the bone-cement connection layer was simulated at 2 mm. In the 6.10-1 Abaqus (INUS Co. rapid form 2004 PP2) program, the three-dimensional model of the bone defect was divided into three-dimensional models; the other structures were tetrahedral elements, except for the bone-cement layer, which was a shell element, and the grid partition of each component was obtained. The total grid number of the metal model was 344,934, and the total number of nodes was 73,683. The total number of the polyethylene model was 262,457, and the total number of nodes was 54,888. A complete summary of the material properties of the bone and implant components is shown in Table 1 [15–17].

**Table 1:** Values of bone and tumor knee prosthesis components

Material	Elasticity(MPa)	Poisson’s ratio	Fatigue resistance(MPa)
Ti-6Al-4 V alloy	110,000	0.30	Yield strength 1010; tensile strength 1080
Vitallium	210,000	0.30	Yield strength 574; tensile strength 736
Cortical bone	13,700	0.30	Compressive strength 55; tensile strength 124
Cancellous bone	1850	0.30	
Bone cement	2070	0.35	Yield strength 44; compressive strength 67
Polyethylene	1070	0.41	Yield strength 21; tensile strength 34

### Experimental conditions and model mechanical loading

The experimental conditions were as follows. First, the prosthesis, bone cement, and bone were all defined as isotropic continuous linear elastic materials. Second, the prosthesis bone cement-bone interface was fully integrated, i.e., relative sliding did not occur under the condition of stress loading. Mechanical loading used a single proximal femoral load, and the force direction was along the femoral axis to the knee joint such that loading was along the longitudinal axis of the femur. Loading mode one was as follows: simulations of a gait with one foot in the upright position with the knee-joint force 2.5 times that of the body weight (2.5 × 650 N = 1600 N) [18]. Loading mode two was as follows: Morrison classic loading curve [19], a knee flexion of 15° with a loading of 2200 N. The mechanical loading method was 8 points, which were selected from the cross section of the proximal femur. The stress magnitude was determined (e.g., 1600 × 1/8 = 200 N; 2200 × 1/8 = 275 N). The boundary constraint condition was complete restraint in the specified loading mode of the distal tibia.

## Results

**Fig. 3:**
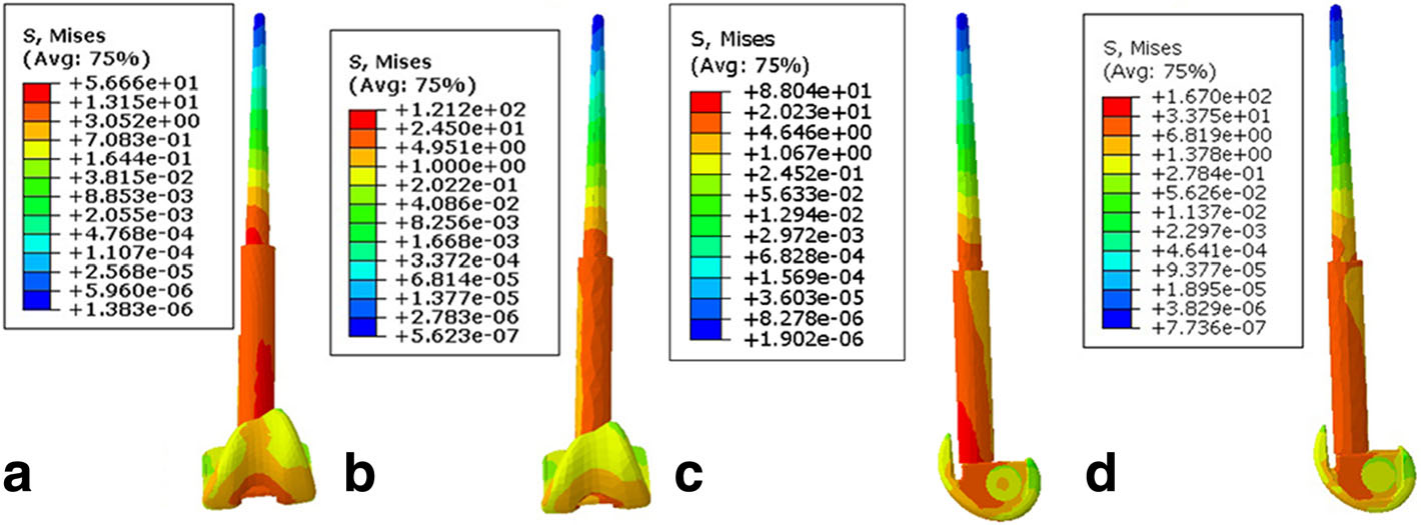
**a**–**d** The overall stress analysis of the femoral stem in the two prosthesis types. **a**, **c** The overall stress analysis of the femoral stem in the metal prosthesis when loading 1600 and 2200 N. **b**, **d** The overall stress analysis of the femoral stem in the polyethylene prosthesis when loading 1600 and 2200 N

The overall stress distribution of the stem of the two types of prostheses was similar. The stress, which was 12–31 MPa, was concentrated on the junction of the stem and shaft. The stem stress from the base to the tip displayed ladder-type dispersion decreases (Fig. 3).

**Fig. 4:**
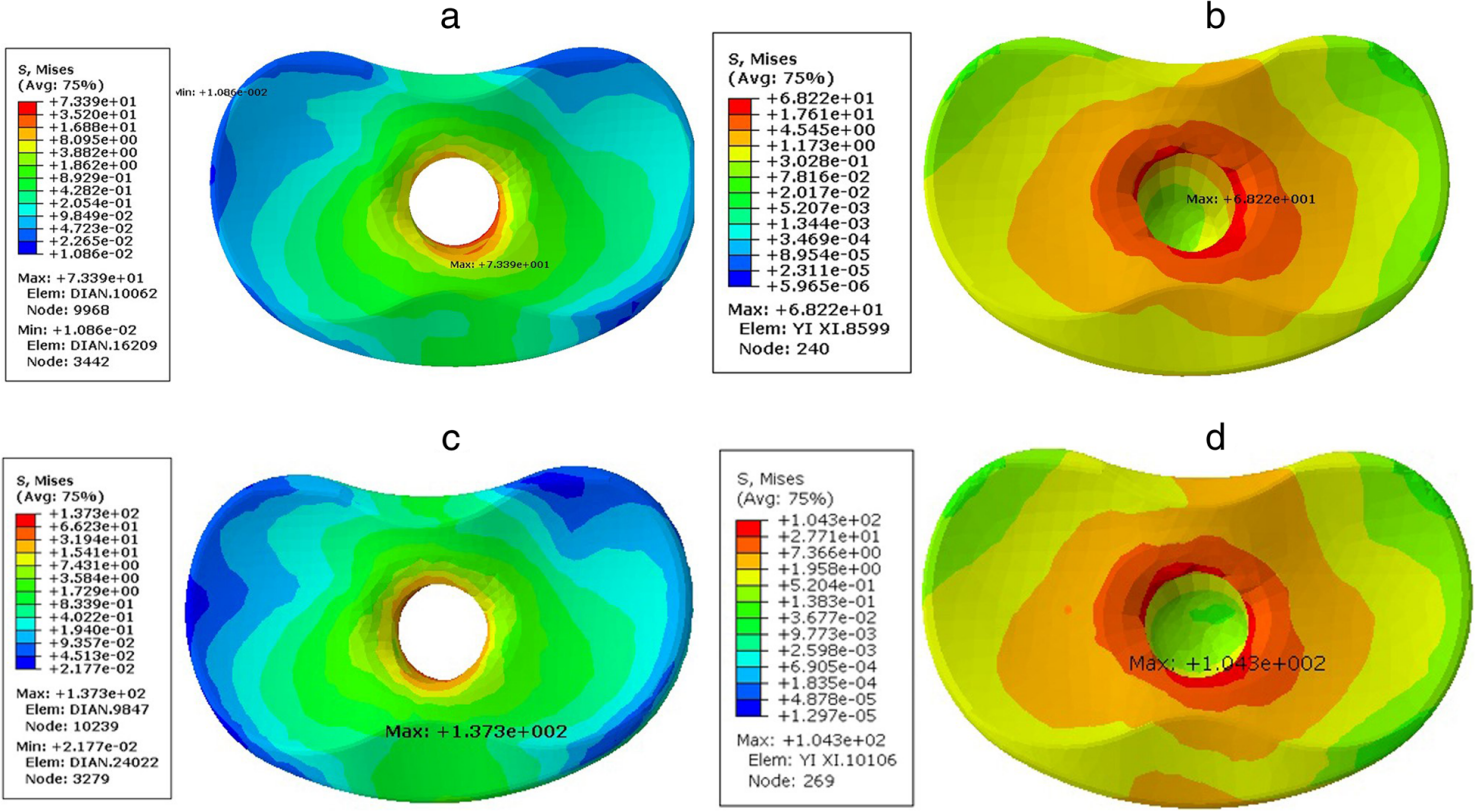
**a**–**d** Stress distribution of the interface at the tibial surface of the two prosthesis types. **a** Stress distribution of the interface at the tibial surface of the MBT prosthesis when loading 1600 N. **b** Stress distribution of the interface at the tibial surface of the APT prosthesis when loading 1600 N. **c** Stress distribution of the interface at the tibial surface of the MBT prosthesis with 15° of knee joint flexion and loading of 2200 N. **d** Stress distribution of the interface at the tibial surface of the APT prosthesis with 15° of knee joint flexion and loading of 2200 N

The von Mises stress on the two types of prostheses at the surface of the tibia was relatively distributed (1–19 MPa), and there was no obvious stress concentration area (Fig. 4). The stress on the outer platform was slightly higher than that on the medial condyle and medial plateau. The stress distribution at the entrance of the femoral medial axis inter surface was 4–137 Mpa, and the MBT was slightly higher than the APT prosthesis.

**Fig. 5:**
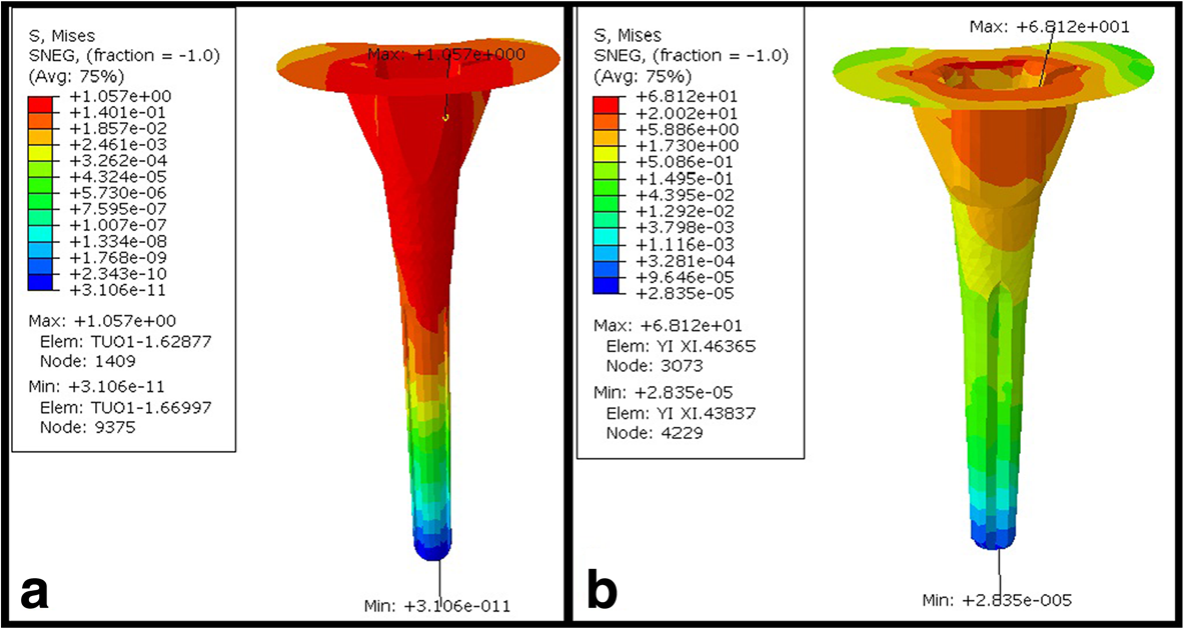
**a**, **b** Stress distribution of the bone-cement for the tibial side of the two prosthesis types with 15° of knee joint flexion and loading of 2200 N. **a** Stress distribution of the bone-cement for the tibial side of the metal prosthesis and **b** stress distribution of the bone-cement for the tibial side of the polyethylene prosthesis

**Fig. 6:**
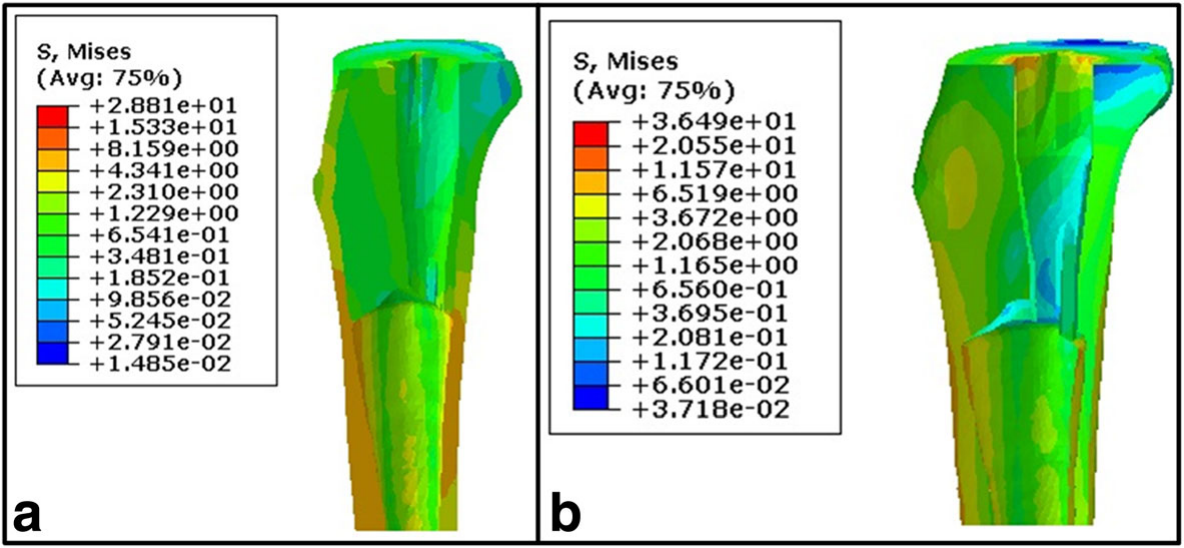
**a**, **b** Stress distribution of the tibia cancellous for the two prosthesis types when loading 1600 N. **a** Stress distribution of the tibia cancellous after the metal prosthesis reconstruction and **b** stress distribution of the tibia cancellous after the polyethylene prosthesis reconstruction

This shear stress distribution was similar on the tibial bone cement layer of the tibial side for both types of prostheses, and the entire high stress concentration area was in the upper part of the bone cement sheath. The shear stress on the tibial bone cement layer of the polyethylene was approximately 20 times higher than that on the metal in the same region (Tables 2 and 3; Fig. 5). The shear stress distribution of the APT prosthesis at the proximal cancellous and cortical bones was 3–5 times higher than that of the MBT type and was relatively more uniform than the MBT. Additionally, there was no obvious stress concentration region (Tables 2 and 3; Fig. 6).

**Table 2:** Maximum stress (MPa) of the two types of prostheses when loading 1600 N

Component	APT prosthesis	MBT prosthesis
Femur stem	24.50	28.89
Femoral condyle	16.06	14.26
Tibial plateau	19.21	16.88
Tibia component	68.22	167.4
Bone cement of the tibia side	8.72	0.54
Tibia bone	11.57	4.34

*APT* all-polyethylene tibia, *MBT* metal-backed tibia

**Table 3:** Maximum stress (MPa) of the two types of prostheses with 15° of flexion of the knee joint and loading 2200 N

Component	APT prosthesis	MBT prosthesis
Femur stem	34.25	37.64
Femoral condyle	19.54	20.72
Tibial plateau	27.71	26.87
Tibia component	104.3	270.9
Bone cement of the tibia side	20.02	1.06
Tibia bone	32.64	13.71

*APT* all-polyethylene tibia, *MBT* metal-backed tibia

## Discussion

The relatively high stress concentration was located at the junction of the stem and shaft with a ladder-like decrease to the end of the handle. No stem breakage occurred in our case series [14]. However, the rate of prosthetic stem breakage was approximately 6–24% and an important cause of early failure of limb salvage [20, 21]. Prosthetic stem breakage was related to the design of the prosthesis, quality of the material, thickness of the stem, and stress on the prosthesis [22]. According to the analysis of the fatigue damage, cracks usually occurred at the location of the concentrated stress, and the material endurance limit was reduced, which was largely due to the effects of the stress concentration [23]. In our study, the concentration of stress was obvious at the junction of the stem and shaft. As a result, the possibility of fatigue fractures of the prosthesis was relatively high over an extended period with long-term use of the knee joint in daily life. As the more intense contact surface of the two types of constructions changed, there was a more obvious stress concentration [24]. This phenomenon can be exploited to reduce the stress concentration.

The stress distributions on the tibial articular surface were essentially the same for the two types of prostheses. High stress was mainly concentrated on the entrance of the femoral shaft. The stress of the outer platform was slightly higher than that of the inner part of the tibial plateau, which indicated that these parts were the main areas of wear in the process of knee flexion and extension. The high stress state of the knee in a normal walking gait may result in serious wear of the tibial prosthetic polyethylene liner, and the potential for a fatigue crack is high. However, a large surface for contact between the intercondylar eminence and polyethylene platform makes the high stress gradually spread to the surrounding area. Therefore, except at the highest stress node, the polyethylene platform in most regions of the stress distribution followed behind the polyethylene yield strength. It appears that the APT component has a thicker high molecular weight polyethylene and reduced one-wear interface, which is more durable than the MBT component. Conventional aseptic loosening manifests as osteoclast-mediated peri-prosthetic resorption of bone, which is likely because microscopic polyethylene debris occurs after interface wear between the metal bottom bracket and polyethylene, which usually presents several years after the original reconstruction surgery [25]. In the most recent aseptic loosening that was treated with revision surgery 7 years after the initial surgery, we found that the APT component showed no obvious wear (Fig. 1). Our goal is to find a polymer material with a slightly higher elastic modulus that could provide similar or greater biocompatibility and better avoid material wear.

In our study, the polyethylene prosthesis bone cement stress value was approximately 20 times higher than that of a metal prosthesis, and the stress was higher in the flexed position. No tibial component failure occurred in our case series. The thickness of bone cement was 2 mm in one study. A previous study did not find a relationship between the thickness of the cement layer and size of the load [26]. Previous studies have confirmed that bone cement is an intact module that connects the prosthesis and bone to distribute stress. However, in the event of a crack, stress will be redistributed, and the failure of the bone cement can accelerate further failure [25, 26]. Cracks in the bone cement may be followed by fatigue fracture, prosthesis loosening, or other complications. In this study, the bone cement layer stress in the metal prosthesis was relatively low. However, although the polyethylene prosthetic bone cement was generally in a high stress state, this was lower than its fatigue endurance limit [15]. Cement stress in other studies also reported increased compressive stress at the cement–cancellous bone interface for the APT implant [17].

The stress distribution of the APT prosthesis at the proximal cancellous bone and cortical bone was 3–5 times higher than that of the MBT prosthesis. The stress was relatively more uniform than the MBT implant, and there was no obvious region of stress concentration. The elastic modulus in the titanium alloy–bone cement–cortical bone interface of the metal prosthesis is largely different, and the stress-shielding effect is inevitable. According to Wolff’s law, when the bone is reconstructed, bone is dissolved and bone mineral loss causes osteoporosis [27]. Van-Lenthe et al. [28] used a three-dimensional FE model to verify that this stress shielding can lead to bone resorption around the prosthesis. As the difference in the elastic modulus of the polyethylene-bone cement and bone in the APT prosthesis is relatively small, the three components can be better integrated with the connecting function of the bone cement to share the stress so that the tibial bone can also gain additional protection. This study outcome was similar to that seen in TKA surgery. Thompson et al. found that higher stress shielding (resorption) occurred around the keel and stem of the MBT, which revealed greater potential for bone loss in these areas. The APT implant had no areas of bone resorption (increased flexible resulted in less stress shielding) [17]. A similar outcome was also found by Scott et al., who reported that significant stress shielding is found in MBT cases, while increased bone density was found in APT cases, particularly in the bones immediately beneath the baseplate. The effect of stress shielding is somewhat reduced for the MBT components compared with the neutral case in the misaligned positions. In APT cases, the effect of stress shielding is mostly low, except in the varus position, which is possibly due to off-loading of the lateral condyle [29].

We acknowledge that the present study has limitations. First, the FE model was based on the anatomy of a single patient. Second, the role of muscles or ligaments was not examined because of the difficulty in assessing the soft tissue changes after excision and reconstruction of the distal femur. Third, we adopted a static-loading simulation dynamic process, which is not the most advanced dynamic loading analysis. Finally, anatomical variations in the distal femur and the extent of excision may affect the results. We hope to address these limitations in future studies.

## Conclusions

Fatigue fracture seems most likely to occur in the stress concentration sites at the junction of the prosthetic femoral shaft and the stem or base of the central axis. The MBT component prosthesis had a powerful stress-shielding effect, which may result in osteolysis of the proximal tibia and subsequent implant failure. In the APT prosthesis, although the stress of the bone-cement layer is relatively high, the stress seems better distributed at the polyethylene–cement–bone interface, which effectively protects the proximal tibia. The results of our study should be considered when selecting the appropriate tibial component for limb-salvage surgery, especially under the foreseeable conditions of osteoporosis.

## Abbreviations

APT: All-polyethylene tibial
CT: Computerized tomography
DICOM: Digital imaging and communications in medicine
FE: Finite element
MBT: Metal-backed tibial
TKA: Total knee arthroplasty
